# Crosstalk between Mitochondrial Protein Import and Lipids

**DOI:** 10.3390/ijms23095274

**Published:** 2022-05-09

**Authors:** Juliane J. Hoffmann, Thomas Becker

**Affiliations:** Institute for Biochemistry and Molecular Biology, Faculty of Medicine, University of Bonn, 53115 Bonn, Germany; juliane.hoffmann@uni-bonn.de

**Keywords:** mitochondria, phospholipids, protein import, TOM complex, SAM complex

## Abstract

Mitochondria import about 1000 precursor proteins from the cytosol. The translocase of the outer membrane (TOM complex) forms the major entry site for precursor proteins. Subsequently, membrane-bound protein translocases sort the precursor proteins into the outer and inner membrane, the intermembrane space, and the matrix. The phospholipid composition of mitochondrial membranes is critical for protein import. Structural and biochemical data revealed that phospholipids affect the stability and activity of mitochondrial protein translocases. Integration of proteins into the target membrane involves rearrangement of phospholipids and distortion of the lipid bilayer. Phospholipids are present in the interface between subunits of protein translocases and affect the dynamic coupling of partner proteins. Phospholipids are required for full activity of the respiratory chain to generate membrane potential, which in turn drives protein import across and into the inner membrane. Finally, outer membrane protein translocases are closely linked to organellar contact sites that mediate lipid trafficking. Altogether, intensive crosstalk between mitochondrial protein import and lipid biogenesis controls mitochondrial biogenesis.

## 1. Introduction

Mitochondria emerged from an endosymbiotic event two billion years ago. A prokaryotic cell was incorporated by an eukaryotic ancestor cell and eventually developed to today’s mitochondrion. Over the course of evolution, the genetic information of the endosymbiont was either lost or transferred to the host genome. The endosymbiotic roots of mitochondria are important for their shape, propagation and functions within the cell. Mitochondria contain two membranes, the outer and inner membranes, as well as the intermembrane space and matrix. The inner membrane forms large invaginations, which are termed cristae and harbor respiratory chain complexes. Mitochondria are essential organelles that perform several biochemical functions for the cell, such as ATP production via oxidative phosphorylation, synthesis of co-factors such as iron-sulfur clusters or coenzyme Q, steps of urea cycle and heme biosynthesis, and biosynthesis of lipids and amino acids. The mitochondrial surface constitutes a signaling platform for programmed cell death, apoptosis and inflammation in innate immunity [1,2]. Dysfunctions of mitochondria have been linked to several diseases, including neurodegenerative disorders [3,4,5].

Mitochondrial functions and biogenesis depend on a large set of proteins. They contain about 900 proteins in baker’s yeast *Saccharomyces cerevisiae* and about 1100 proteins in humans [5,6,7,8,9]. Due to their endosymbiotic origin, mitochondria harbor their own genomes. However, this circular DNA encodes just for a small number of proteins: 8 proteins in yeast and 13 proteins in human mitochondria. About 99% of mitochondrial proteins are produced as precursors on cytosolic ribosomes. The precursor proteins contain cleavable or non-cleavable targeting signals that mediate their targeting to the mitochondrial surface [10,11]. The translocase of the outer membrane (TOM complex) imports the vast majority of mitochondrial proteins. Subsequently, membrane-bound multisubunit protein translocases sort the incoming precursor proteins into the outer and inner membrane, intermembrane space and matrix [12,13,14,15,16,17].

Mitochondrial functions also depend on the import and biosynthesis of phospholipids. The phospholipid composition of mitochondrial membranes is critical for several different processes, including mitochondrial dynamics, apoptosis, oxidative phosphorylation and membrane architecture for a more detailed overview [18,19,20,21,22]. Mitochondrial membranes contain the characteristic phospholipid cardiolipin (CL). The capability of mitochondria to produce CL is a relic of their endosymbiotic origin. Defects of biosynthesis of CL have been associated with cardiovascular diseases [23,24,25]. We propose that there is a close link between mitochondrial protein import and lipid biosynthesis. Protein translocases are linked to organelle contact sites that mediate lipid trafficking between mitochondria and other cellular compartments. Furthermore, the phospholipid composition of mitochondrial membranes also affects protein import into the mitochondria. Phospholipids are important for the integrity and functions of protein translocases, as well as the maintenance of the mitochondrial membrane potential that drives protein transport across and into the inner membrane. Structural characterizations of protein translocases have revealed how phospholipids affect protein transport. We focus on recent structural and biochemical data to point out the role of lipids in protein import and highlight the crosstalk between protein and lipid biogenesis.

## 2. Mitochondrial Phospholipids

**Figure 1:**
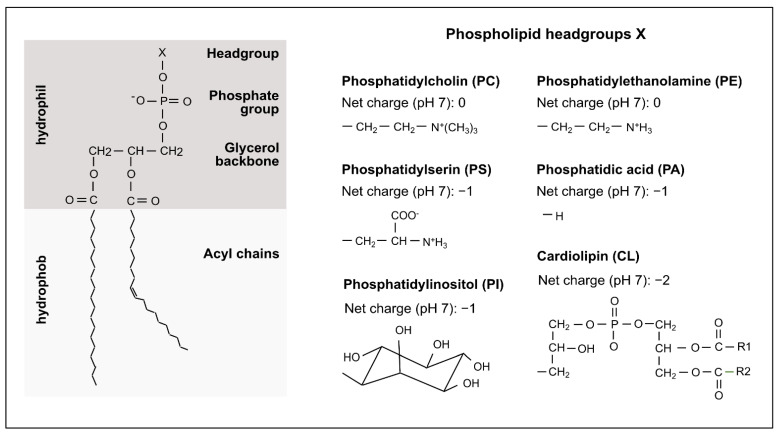
Mitochondrial phospholipids. Phospholipids contain a hydrophilic head with a glycerol backbone as the central structural element and distinct headgroups, each linked by a phosphate group. The headgroup is characteristic for each class of phospholipids: phosphatidylcholine (PC), phosphatidylethanolamine (PE), phosphatidylinositol (PI), phosphatidylserine (PS), phosphatidic acid (PA) and cardiolipin (CL). It varies in its net charge and is linked via a phosphate group to glycerol. Acyl chains are also attached to the glycerol backbone via ester bonds and form the hydrophobic tail of the phospholipids. The acyl chains can vary in length and saturation. Figure 1 is adapted from [19].

Mitochondrial membranes in yeast contain about 40–45% phosphatidylcholine (PC), 25–30% phosphatidylethanolamine (PE), 10–15% of phosphatidylinositol (PI), 10–15% CL, 3–5% phosphatidylserine (PS) and up to 2% phosphatidic acid (PA) [26,27]. Sphingolipids and sterols are present only in low amounts [27]. The lipid composition of the outer and inner membranes differs strongly. Whereas the outer membrane has a low protein content, the inner membrane is the most protein-enriched membrane in the cell [26,27]. Furthermore, CL is strongly enriched in the inner membrane, whereas it is a low-abundant lipid in the outer membrane [28]. The charge of the headgroup and the fatty acid chain composition are important features of phospholipids that can affect the functions of protein machineries (Figure 1). PI, PS, PA and CL have a negatively charged headgroup, whereas the most abundant phospholipids, PE and PC, possess a neutrally charged headgroup. Mitochondria are rich in so-called non-bilayer phospholipids, such as PE and CL. The headgroup of these lipids has a smaller diameter than the fatty acid tail. Depending on pH and cation concentrations, isolated non-bilayer phospholipids form non-lipid bilayer structures [26,29,30]. In contrast, the headgroup and the fatty acid domain of bilayer-forming phospholipids like PC, PI and PS have a similar diameter. Non-bilayer phospholipids affect membrane curvature and locally destabilize the lipid bilayer, which are both important features for the function of membrane-bound protein machineries [26,29]. The presence of non-bilayer lipids is critical for mitochondrial functions. Supporting this view, the parallel loss of CL synthase and mitochondrial PE synthesis is lethal [31].

Mitochondria synthesize the non-bilayer phospholipids CL and PE. CL is produced at the mitochondrial inner membrane in a multistep pathway. The precursor PA is transported from the endoplasmic reticulum (ER) to the mitochondrial inner membrane, where the CDP-diacylglycerol (DAG) synthase Tam41 catalyzes the reaction of PA with cytidine phosphate to CDP-DAG [32]. Pgs1 combines glycerol-phosphate with CDP-DAG to produce phosphatidylglycerolphosphate (PGP). Subsequent removal of phosphate leads to the production of phosphatidylglycerol (PG). The CL synthase Crd1 adds a second CDP-DAG to PG to form CL. The produced CL undergoes remodeling of its acyl chain composition by the sequential activity of the CL-specific deacylase Cld1 and the monolyso-acyltransferase Taz1, termed Taffazin, in humans [20,23,24,25,26,27,33]. Mutations of human Taffazin have been found in patients suffering from Barth syndrome, which is characterized by dilated cardiomyopathy, neutropenia, growth retardation and 3-methyl-glutaconic aciduria [20,23,24,25,26,27,33].

Mitochondrial PS decarboxylase 1 (Psd1) decarboxylates PS to produce the majority of cellular PE in yeast. Additional pathways for the production of PE exist [27,34]. First, ethanolamine is activated in a two-step reaction to CDP-ethanolamine, which reacts with DAG to form PE. In human cells, the Kennedy pathway typically represents the dominant pathway to generate PE. Second, the vacuolar Psd2 decarboxylates PS to form PE in the vacuolar membrane. Third, the acyltransferases Tge3 and Ale1 generate minor amounts of PE [27,34]. The biogenesis of Psd1 involves autocatalytic cleavage into the membrane anchored β-subunit and the catalytic α-subunit. This autocatalytic cleavage is critical for activating the enzyme. Both subunits remain stably bound to each other to allow PE biosynthesis on the intermembrane space side of the inner membrane [35,36]. Other phospholipids such as PC, PS, PA and PI are produced in the ER membrane and have to be imported into mitochondria. Organelle contact sites, such as the ER mitochondria encounter structure (ERMES) or vacuole mitochondria patch (vCLAMP), play an important role in the exchange of phospholipids between mitochondria and other cellular compartments [37,38,39,40].

## 3. Overview of Mitochondrial Protein Import Pathways

Mitochondrial precursor proteins are produced on cytosolic ribosomes. According to our current knowledge, protein import into mitochondria occurs predominantly in a post-translational manner [10,11]. Cytosolic factors guide precursor proteins to the mitochondrial surface. Proteins are imported in a largely unfolded stage to allow the passage of the translocation channel. The TOM complex is the general entry gate for most precursor proteins. The β-barrel protein Tom40 forms the protein-conducting channel [41,42,43,44,45]. All other TOM subunits contain a single α-helical membrane anchor. Structural analyses of yeast and human TOM complexes revealed a highly similar molecular organization, including two Tom40 molecules linked via two Tom22 subunits. Each Tom40 is associated with three small Tom proteins, Tom5, Tom6 and Tom7 [46,47,48,49,50]. This TOM core complex associates with the receptors Tom20 and Tom70, which recognize different types of incoming precursor proteins [51,52,53,54,55]. The cytosolic domain of Tom22 forms the docking site for both receptor proteins in the TOM complex [56].

**Figure 2:**
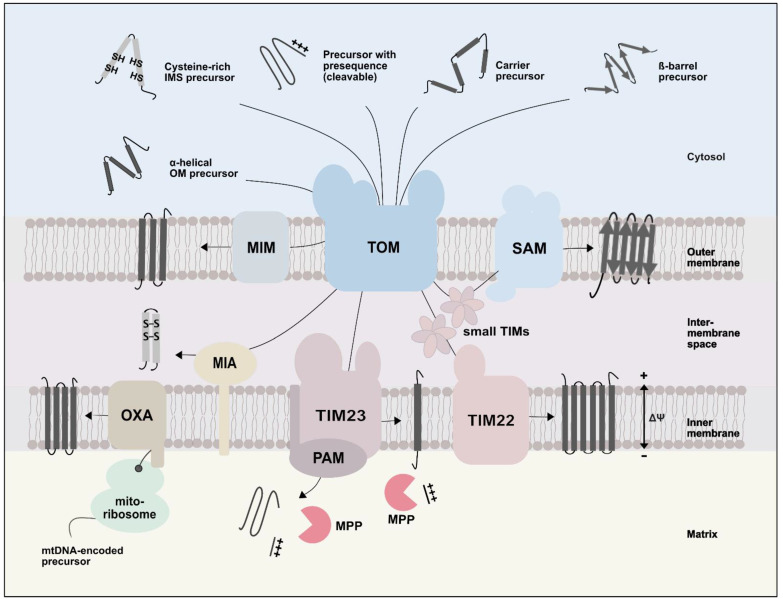
Protein import pathways into mitochondria. Mitochondria have to import the majority of their proteins, which are synthesized as precursors on cytosolic ribosomes. The translocase of the outer membrane (TOM) forms the general entry gate for mitochondrial precursor proteins. After passing the TOM channel, β-barrel precursors are guided by small TIM chaperones of the intermembrane space to the sorting and assembly machinery complex (SAM), which mediates their folding and insertion into the outer membrane. The presequence translocase of the inner membrane (TIM23) transports precursors with a cleavable presequence into and across the inner membrane. Translocation into the matrix depends on the ATP-depending activity of the presequence translocase-associated motor (PAM). After import, mitochondrial processing peptidases (MPP) remove the cleavable presequence. In the carrier pathway, small TIM chaperones transfer the hydrophobic carrier precursor from the TOM complex to the carrier translocase (TIM22), which inserts them into the inner membrane. The membrane potential (Δψ) drives protein import into and across the inner membrane. The mitochondrial intermembrane space import and assembly machinery (MIA) directs cysteine-rich precursors into the IMS and mediates their oxidative folding. The mitochondrial import machinery (MIM complex) inserts precursors of single and multi-spanning proteins into the OM. Finally, the oxidase assembly machinery (OXA) inserts mitochondrially encoded proteins into the mitochondrial IM.

After passage of the TOM complex, specific protein translocases sort the precursor proteins into the outer and inner membranes, the intermembrane space, and the matrix (Figure 2) [12,13,14,15]. First, the presequence translocase (TIM23 complex) transports precursor proteins containing a cleavable presequence into the matrix and inner membrane. Second, the carrier translocase (TIM22 complex) integrates proteins with several transmembrane segments, such as carrier proteins, into the inner membrane. Small TIM chaperones guide these hydrophobic precursor proteins toward the TIM22 translocase. The activity of the respiratory chain generates a membrane potential across the inner membrane, which drives protein transport via the presequence and carrier translocase [57,58]. Third, Mia40 (mitochondrial intermembrane space import and assembly machinery) oxidatively folds cysteine-rich proteins, which trap them in the intermembrane space. Fourth, β-barrel precursors are transported from the TOM complex to the sorting and assembly machinery (SAM complex, also termed the TOB complex for topogenesis of β-barrel proteins), which inserts them into the outer membrane. Finally, the mitochondrial import (MIM) machinery inserts proteins with α-helical membrane anchor into the outer membrane [12,13,14,15]. The MIM complex has so far only been identified in fungi. A functional paralog was described in *Trypanosoma brucei* [59], but the human equivalent awaits identification. OXA1 (oxidase assembly) integrates mitochondrially encoded proteins into the inner membrane [12,13,14,15]. OXA1 also promotes the insertion of some nuclear encoded precursor proteins into the inner membrane [60,61,62].

## 4. Role of Lipids in Protein Import into Mitochondria

**Figure 3:**
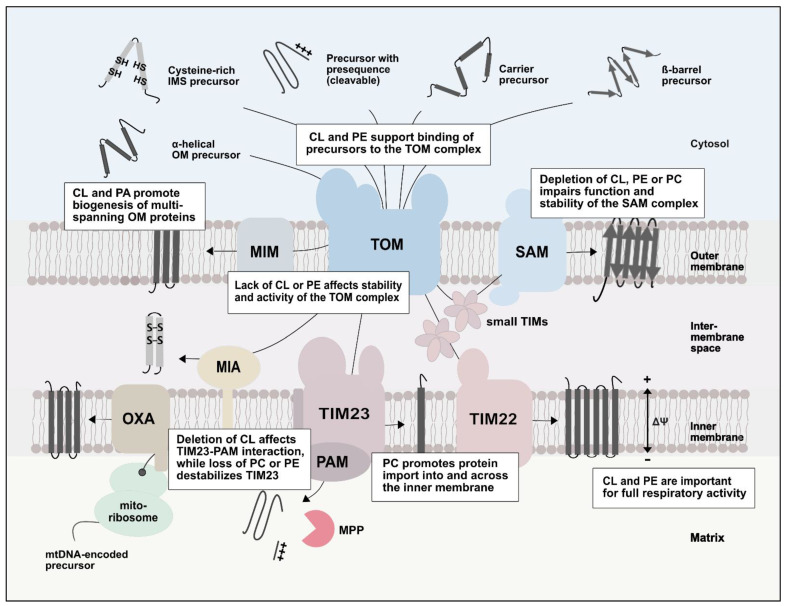
Role of phospholipids in mitochondrial protein import. Alterations in the composition of phospholipids of the mitochondrial membranes affect protein import pathways and protein translocases at different levels. The phospholipids CL, PE and PC are important for the stability and function of outer and inner membrane protein translocases. The depletion of CL or PE affects the respiratory chain, which causes a reduction of the membrane potential and impaired protein translocation across and into the inner membrane. Figure 3 is adapted from [19].

Protein import into mitochondria is modulated by changes in the phospholipid composition of the mitochondrial membranes (Figure 3). The phospholipid composition is important for the stability and operation of protein translocases and has an impact on dynamic protein–protein interactions. Phospholipids affect the activity of respiratory chain complexes, which in turn leads to reduced membrane potential and impaired protein import. The combination of structural and biochemical studies provides first insights into how phospholipids affect mitochondrial protein transport on a molecular level.

### 4.1. Role of Lipids in the TOM Complex

The TOM complex forms the entry gate for the majority of mitochondrial precursor proteins and is therefore essential for life. The central β-barrel protein Tom40 is associated with Tom22 and the small Tom proteins, which all contain a single α-helical membrane anchor [46,47,48,49,50]. Two Tom22 subunits link two Tom40 subunits and are critical for the integrity of the translocase. Remarkably, the structural analyses revealed the presence of a phospholipid between Tom40 and Tom22, which could be PE in yeast or PC in humans [47,48,49,50]. A structure of the human TOM complex revealed the presence of further phospholipids, such as PE and PC, which were detected between Tom40 and Tom6, Tom7 and Tom22 [50]. These data indicate that phospholipids could play an important role in the integrity and function of the TOM complex. Indeed, the binding of a model precursor protein to the TOM complex is impaired in PE-deficient yeast mitochondria [63,64]. Supporting this view, the transport of β-barrel proteins across the outer membrane is decreased in PE-deficient mitochondria, whereas TOM-independent protein import remains unaffected [63]. The binding of precursor proteins to the TOM complex is similarly affected in the absence of CL in yeast mitochondria [28]. Thus, non-bilayer-forming phospholipids are important for the function of the TOM complex. In contrast, depletion of the bilayer-forming PC does not interfere with precursor binding to the translocase [64]. However, the molecular mechanisms by which non-bilayer phospholipids affect the binding of precursor proteins to the TOM complex remains unknown. CL also stabilizes the association of Tom20 with the TOM complex [28]. Structural data of the TOM complex containing Tom20 and Tom70 are missing. Therefore, it remains enigmatic how CL affects the docking of Tom20 to the TOM complex.

### 4.2. Effects of Lipids on the Import of β-Barrel Proteins

Yeast mitochondria contain five β-barrel proteins that are essential for the transport of proteins (Tom40, Sam50, Mdm10) and metabolites and ions (Porin isoforms: Por1 and Por2) across and into the outer membrane and are part of organelle contact sites (Mdm10). Precursors of β-barrel proteins are first transported across the outer membrane via the TOM complex and subsequently integrated into the outer membrane by the SAM complex [13,14,15,65]. TOM and SAM complexes form a supercomplex to facilitate the transfer of precursor proteins [66,67]. The small TIM chaperones stabilize the precursor at the TOM-SAM complex [66,67,68,69,70]. Structural and biochemical analyses of the past few years have provided important insights into the operation of the SAM complex on a molecular level [71,72,73]. The SAM complex contains two peripheral subunits, Sam37 and Sam35, and the β-barrel protein Sam50. Sam50 belongs to the Omp85 protein family, which is present in the outer membrane of bacteria, mitochondria and plastids from plants [74,75,76]. It contains one polypeptide translocation-associated domain (POTRA) that faces the intermembrane space and a 16-stranded transmembrane β-barrel. The β-barrel of Sam50 forms a lateral gate between the first and the last β-strands. Hairpins of two β-strands of the incoming precursor protein are inserted sequentially into the lateral gate, leading to the stepwise formation of the β-barrel [77]. The short β-strands of the lateral gate induce thinning and distortion of the lipid bilayer to facilitate insertion of the growing β-barrel [71,72]. The maturation and release of the newly formed β-barrel proteins involves a β-barrel switching mechanism [72]. In the idle state, the SAM complex contains two Sam50 monomers. The second Sam50 functions as a placeholder to facilitate the insertion and folding of the β-barrel precursor protein. The insertion of a β-barrel precursor into the lateral gate of the Sam50 displaces the second Sam50 to allow the new β-barrel to form [72]. Finally, the efficient release of the Tom40 precursor from the SAM complex depends on the binding of Mdm10 to the SAM complex [72,78,79].

Altogether, the formation of β-barrel proteins at the SAM complex involves massive molecular rearrangements at the protein translocases, such as coupling of partner proteins and formation of β-barrel that occurs within the lipid bilayer. Therefore, it is not surprising that protein sorting via the SAM complex strongly depends on the native phospholipid composition of the outer membrane. Import of β-barrel proteins is impaired in yeast mitochondria deficient of either CL, PE or PC [28,63,64]. Inspection of different biogenesis steps of Tom40 revealed that CL and PE are particularly important for the initial binding of the precursor to the SAM complex [28,63]. Interestingly, two phospholipids were found in the interface between the two Sam50 molecules and between Sam50 and Mdm10 [72]. However, their identity remains unknown. We speculate that these phospholipids may affect the molecular dynamics of the SAM complex. Future studies are required to define the biophysical properties of phospholipids or the membrane environment that are important for protein transport via SAM machinery.

The SAM complex also constitutes a platform for the assembly of the Tom40 precursors with other TOM subunits. Biochemical and structural data revealed that Tom5 and Tom6 assemble with Tom40 at the SAM complex [73,80,81,82]. Furthermore, the SAM-Mdm10 complex promotes the insertion and assembly of the Tom22 precursor [78,80,83]. The assembly of Tom22 with Tom40/small Tom modules is a critical step in the formation of a mature TOM complex [56]. The assembly of these single-spanning TOM subunits is defective in PC-deficient yeast mitochondria [64], whereas depletion of neither CL nor PE impairs their assembly steps [28,63]. We propose that the important role of phospholipids in the assembly of the TOM complex could be explained by the presence of lipids in the interface between the β-barrel of Tom40 and further TOM subunits [47,48,50]. Altogether, the activity of the dynamic SAM complex in protein biogenesis strongly depends on the proper lipid composition of the outer membrane.

### 4.3. Lipids and the Import of α-Helical Outer Membrane Proteins

The outer membrane contains several proteins with single or multiple α-helical transmembrane segments. Different import mechanisms involving TOM, SAM subunits or protein-independent insertion have been reported [13,14,15,65]. The MIM complex is the major protein translocase for single and multi-spanning proteins in yeast mitochondria [84,85,86,87,88,89,90,91,92]. It is a highly dynamic protein machinery that cooperates with different partner proteins, such as the SAM and TOM subunits [84,85,86,87,88,89,90,91,92]. Therefore, we speculate that the lipid environment may be important for MIM-dependent protein import. Indeed, negatively charged phospholipids CL and PA promote the import of multi-spanning proteins [93,94]. The non-bilayer forming properties of these lipids seem not to be critical since depletion of the most abundant non-bilayer phospholipid PE does not affect import of these proteins [63]. Finally, high sterol content impairs the integration of the C-terminally anchored Fis1 into the outer membrane [95,96]. The molecular mechanisms of the MIM-dependent protein insertion into the outer membrane remain to be determined. Therefore, it is unclear how lipids affect this import pathway at the molecular level.

### 4.4. The Role of Lipids in Inner Membrane Protein Transport Processes

The inner mitochondrial membrane harbors two major protein translocases, the TIM23 translocase, which sorts presequence-containing proteins, and the TIM22 complex, which integrates carrier proteins into the inner membrane. The membrane potential is critical for driving protein import via both protein import pathways [57,58]. Respiratory chain complexes transport protons across the inner membrane into the intermembrane space to build up the membrane potential. Several studies have revealed that phospholipids are important for the stability and function of respiratory chain complexes. Different phospholipids have been found in the structures of respiratory chain complexes [97,98]. In yeast, complex III (cytochrome bc1 complex) and complex IV (cytochrome c oxidase) associate in respiratory chain supercomplexes [99,100,101], whereas mammalian complex I (NADH dehydrogenase), complex III and complex IV form high molecular weight supercomplexes [100]. CL stabilizes the association of respiratory chain complexes III and IV into supercomplexes in yeast mitochondria [102,103,104,105,106]. In contrast, depletion of PE does not interfere with structural integrity, but is required for the full activity of respiratory chain complexes [107,108,109,110]. Yeast mutant strains that are defective in PE biosynthesis frequently display reduced membrane potential, which in turn affects protein transport into and across the inner membrane [108]. Depending on the growth conditions, depletion or loss of CL also affects respiratory activity, membrane potential, and consequently protein import [111,112,113,114,115]. In contrast, the activity and stability of respiratory chain complexes are not impaired in PC-deficient cells [110,116]. In our view, phospholipids differentially affect the function and stability of respiratory chain complexes.

Phospholipids are important for the structural integrity of the TIM23 complex and its dynamic coupling of partner proteins. The presequence translocase consists of seven subunits. The essential components Tim23 and Tim17 form a pore that releases proteins into the membrane and promotes protein translocation into the matrix [117,118,119,120]. Mgr2 closely associates with Tim17 and Tim23 and monitors the lateral gate to control the release of membrane proteins into the inner membrane [121]. Tim23 exposes a soluble domain into the intermembrane space that coordinates with Tim50 and Tim21 recognition of incoming precursor proteins and their transfer to the translocation pore [54,122,123,124,125]. Lateral release into the inner membrane depends on the membrane potential as a driving force and is supported by the presence of CL in the membrane [126]. Protein transport into the matrix also involves the dynamic association and ATP-consuming activity of the presequence translocase-associated motor (PAM). Here, Tim44 forms a docking site for mitochondrial Hsp70 that powers via ATP-hydrolysis protein translocation into the matrix. Co-chaperones of the PAM module control the reaction cycle of mitochondrial Hsp70 [127,128,129]. Altogether, the presequence translocase undergoes dynamic molecular switches to promote protein transport into the matrix or inner membrane.

CL stabilizes the TIM23 complex in different ways [115,130,131,132,133]. First, CL supports the interaction of the PAM module with the TIM23 complex [113,115]. Second, the association of Tim44 with membranes is promoted when CL is present [130,131]. Finally, the association of Tim50, Tim21 and Tim17 with Tim23 is destabilized in CL-deficient mutant mitochondria [115,133]. Similarly, the amount and integrity of the presequence translocase are reduced in PC- and PE-deficient mitochondria [108,116]. Remarkably, membrane potential and respiratory activity are only mildly decreased upon the loss of PC, but protein import via the presequence pathway is reduced, indicating a direct role of PC in protein transport via the TIM23 complex [116]. Further structural and detailed biochemical studies should reveal how PC and PE affect TIM23 stability and function.

The carrier translocase integrates multi-spanning proteins into the inner membrane. The central subunit Tim22 inserts proteins into the inner membrane [57]. Other subunits of the TIM22 complex mediate the assembly of the translocase and docking of small TIM chaperones. Defects in the membrane potential are a major cause for reduced import of carrier proteins in PE- and CL-deficient mitochondria [108,111]. Although the membrane potential is intact, the import of carrier proteins is reduced in PC-deficient mitochondria [116], pointing to a more direct role of PC in protein translocation. How phospholipids affect TIM22-mediated protein insertion remains unknown. Recently reported cryo-electron microcopic structures did not reveal phospholipids in the yeast and human TIM22 complex [134,135]. The TIM22 complex remains largely intact in lipid mutant mitochondria [108,114,116], but the assembly of the imported carrier proteins like ADP/ATP carrier depends on the presence of CL [111,114,115,136,137]. Remarkably, acylglycerolkinase (AGK) is a subunit of the human TIM22 complex. AGK is involved in lipid metabolism and phosphorylates monoacylglycerol and diacylglycerol to lysophosphatidic acid and phosphatidic acid, respectively [135,138,139]. Mutations of AGK have been linked to patients suffering from Sengers syndrome [140]. Why the enzyme AGK is a component of the TIM22 complex remains to be clarified. Inactivation of kinase activity does not affect its function in protein import, indicating that the structural properties of AGK are important for its function in protein transport [138,139]. The dual role of AGK might coordinate protein translocation and lipid metabolism in mitochondria.

## 5. Connection of Protein Transport and Organelle Contact Sites

Most phospholipids are produced in the endoplasmic reticulum, while mitochondria synthesize CL and parts of PE [19,26,27,39,40]. Consequently, mitochondrial biogenesis depends on the import of phospholipids from the ER. Mitochondria do not receive lipids via vesicular trafficking, since they are not part of the endomembrane system. Instead, molecular contact sites allow the exchange of lipids with different cellular compartments. In yeast, the ERMES complex mediates lipid trafficking between ER and mitochondria, whereas the vacuole and mitochondria patch (vCLAMP) facilitates the exchange of lipids with the vacuolar membrane [141,142,143,144,145,146,147].

**Figure 4:**
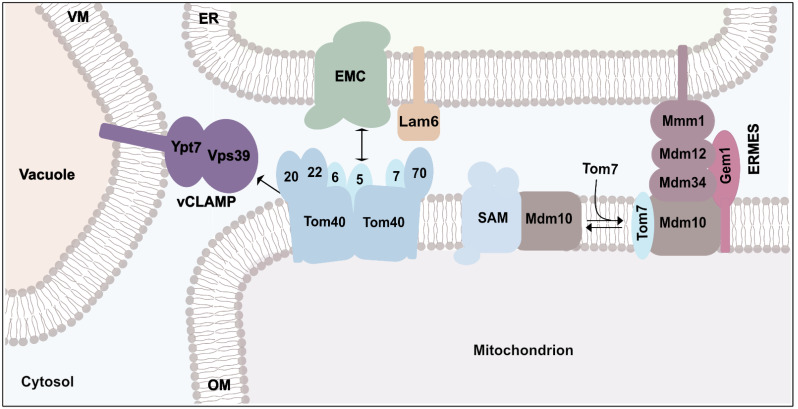
Connections of protein transport and organelle contact sites. Organelle contact sites allow lipid trafficking between mitochondria and other cellular organelles such as the endoplasmic reticulum (ER). The ER–mitochondria encounter structure (ERMES) forms a molecular bridge between ER and mitochondria. It consists of the ER-anchored protein Mmm1, which is linked via Mdm12 and Mdm34 to Mdm10. Gem1 and Tom7 are associated with ERMES. Mdm10 also binds to the SAM complex to promote protein biogenesis. Binding of Tom7 detaches Mdm10 from the SAM complex and promotes its association with ERMES. The ER membrane protein complex (EMC) and ER-localized Lam6 interact with the Tom5 and Tom70 of the TOM complex, respectively. The vacuole and mitochondria patch (vCLAMP) link mitochondria to the vavuolar membrane. vCLAMP consists of the vacuolar membrane (VM) protein Ypt7 and the vacuolar protein sorting 39 (Vps39), which binds to Tom40 to mediate the exchange of lipids between the vacuole and mitochondria.

Remarkably, there are several connections between protein import and organelle contact sites, pointing to a close link between protein and lipid transport [148]. The TOM complex is linked to organelle contact sites (Figure 4). In a split-Venus approach, Tom5 was found as an interaction partner of the ER membrane complex (EMC) and a function of EMC in lipid transport to mitochondria was reported [149]. The molecular mechanism by which lipids are transported via EMC and Tom5 remains to be demonstrated. Interestingly, EMC inserts proteins into the ER membrane [150,151]. How the activities of the EMC complex are coordinated remains unknown. The Tom70 receptor interacts with the sterol transport protein Lam6/Ltc1 of the ER membrane [152]. Lam6/Ltc1 is present at different organellar contact sites between ER, mitochondria and the vacuole and coordinates the formation of ERMES and vCLAMP [153]. Finally, Tom40 is the mitochondrial binding partner of Vps39 in the vCLAMP structure [154]. Whether or not the Tom40 β-barrel is involved in lipid trafficking remains enigmatic.

Mdm10 has dual localization. It is associated with the SAM complex [72,78,79,83,155] and forms the mitochondrial anchor of the ERMES complex [78,156]. Tom7 controls the distribution of Mdm10 between both protein complexes. Binding of unassembled Tom7 dissociates the SAM-Mdm10 complex and promotes the association of Mdm10 with ERMES [78,79,157]. The ERMES complex consists of the synaptotagmin-like mitochondrial lipid-binding protein (SMP) domains containing Mmm1, Mdm12 and Mdm34 and the mitochondrial Mdm10. Such SMP domains have been implicated in mediating lipid transport. Structural data reveal that the ER-resident Mmm1 and Mdm34 form a hydrophobic tunnel that allows lipid transport [145,146]. Interestingly, the inner wall of the Mdm10 β-barrel contains hydrophobic patches [158]. It remains to be demonstrated whether the β-barrel of Mdm10 transports lipids into the outer membrane.

Altogether, outer membrane protein translocases are closely linked to organelle contact sites. The functional implications of these connections remain elusive. We speculate that these molecular interactions are important for coordinating protein and lipid transport during mitochondrial biogenesis and functions.

## 6. Conclusions

Protein import is closely linked to the phospholipid composition of mitochondrial membranes. Remarkably, the individual depletion of the different phospholipid classes has distinct effects on the different protein import routes. Whereas CL and PE are important in maintaining respiratory chain function and membrane potential-dependent import pathways, depletion of PC did not affect the activity of respiratory chain complexes [108,111,116]. CL and PE, but not PC, promote precursor binding to the TOM complex [28,63,64]. We propose that the non-bilayer features of CL and PE are important for the function of the TOM complex. The function of dynamic protein translocases, such as the SAM complex and the TIM23 translocase, is strongly dependent on the native lipid composition [28,63,64,113,115,126]. Structural data reveal that phospholipids are present in the interface of translocase subunits and dynamically associated partner proteins [47,48,50,72]. Studies addressing the impact of the negatively charged PI and PS on protein transport are currently missing and will be important to define which features of lipids are important for their function in protein import. A recent study reported that the knockdown of PS synthase in *Drosophila melanogaster* leads to reduced mitochondrial PS and CL and impaired localization of mitochondria-targeted EYFP [159]. However, whether PS affects mitochondrial protein import on molecular levels remains to be determined. Protein translocases dynamically interact with each other and with several partner proteins to fine-tune protein transport to cellular needs [13,14,15]. The impact of lipids on the organization of such molecular interactions remains to be determined. Lipids are also important for cellular signaling, which can regulate protein import into mitochondria. In human cells, a lipid signaling cascade is initiated upon starvation and hypoxia, which results in the downregulation of the mitochondrial PE content [160]. The decreased PE content in turn stimulates the inner membrane AAA protease YME1L, which degrades inner membrane-bound protein translocases, lipid transfer proteins and metabolic enzymes [160]. Altogether, we propose that protein and lipid biogenesis are closely connected to each other to control mitochondrial biogenesis and function.
